# Characteristics, Occurrence, Detection and Detoxification of Aflatoxins in Foods and Feeds

**DOI:** 10.3390/foods9050644

**Published:** 2020-05-18

**Authors:** Amirhossein Nazhand, Alessandra Durazzo, Massimo Lucarini, Eliana B. Souto, Antonello Santini

**Affiliations:** 1 Department of Biotechnology, Sari Agricultural Science and Natural Resource University, 9th km of Farah Abad Road, Mazandaran 48181-68984, Iran; nazhand.ah@gmail.com; 2 CREA-Research Centre for Food and Nutrition, Via Ardeatina 546, 00178 Roma, Italy; alessandra.durazzo@crea.gov.it (A.D.); massimo.lucarini@crea.gov.it (M.L.); 3 Faculty of Pharmacy of University of Coimbra, Azinhaga de Santa Comba, Polo III-Saúde, 3000-548 Coimbra, Portugal; souto.eliana@gmail.com; 4 CEB-Centre of Biological Engineering, University of Minho, Campus de Gualtar, 4710-057 Braga, Portugal; 5 Department of Pharmacy, University of Napoli Federico II, Via D. Montesano 49, 80131 Napoli, Italy

**Keywords:** aflatoxins, mycotoxins, detoxification, food safety, health issue

## Abstract

Mycotoxin contamination continues to be a food safety concern globally, with the most toxic being aflatoxins. On-farm aflatoxins, during food transit or storage, directly or indirectly result in the contamination of foods, which affects the liver, immune system and reproduction after infiltration into human beings and animals. There are numerous reports on aflatoxins focusing on achieving appropriate methods for quantification, precise detection and control in order to ensure consumer safety. In 2012, the International Agency for Research on Cancer (IARC) classified aflatoxins B1, B2, G1, G2, M1 and M2 as group 1 carcinogenic substances, which are a global human health concern. Consequently, this review article addresses aflatoxin chemical properties and biosynthetic processes; aflatoxin contamination in foods and feeds; health effects in human beings and animals due to aflatoxin exposure, as well as aflatoxin detection and detoxification methods.

## 1. Introduction

Food contamination is a global concern in the stages of the production, distribution and consumption of agricultural and processed products [1,2,3,4,5,6,7,8,9,10]. From the perspective of a joined and integrated approach to food research, three aspects of foods and the food chain should be investigated: quality, safety, and potential nutraceutical value [11,12,13,14,15,16,17,18,19,20,21,22,23]. Food safety is currently a priority in the processes of the production, processing and distribution of food products. Micro-fungi such as *Penicillium*, *Fusarium* and *Aspergillus* that grow on foods and feeds when conditions are suitable, are able to release secondary metabolites (mycotoxins) that endanger the health of humans and animals after being consumed [24,25,26,27,28,29,30,31]. The Centers for Disease Control and Prevention (CDC) reported that approximately 4.5 billion people are chronically exposed to mycotoxins [32]. There are over 300 mycotoxins, the most important of which include aflatoxins (AF), patulin, fumonisins, ochratoxins, ergotamine, deoxyvalenol, and zearalenone [33,34,35,36]. Aflatoxins are the main mycotoxins synthesized by *Aspergillus flavus*, *A. parasiticus* and *A. nomius* [37,38,39]. Aflatoxin-related contamination by fungi can occur in food and feed products (e.g., cocoa, spices, figs, rice, wheat, maize, sesame seeds, millet, and groundnuts) during the processes before and after harvesting [40,41,42,43,44,45,46,47,48,49,50,51,52]. Moreover, AF can contaminate commercial products such as cosmetics, cooking oil, and peanut butter. The Food and Agriculture Organization (FAO) reported that 25% of global food crops can be contaminated by mycotoxins [53]. Although much research has been conducted in this area, AF-related contamination is still a problem in agriculture and human health worldwide [54]. Because of the adverse effects of AFs, these compounds have been included in the European Union’s Rapid Alert and Food Alert System (RASFF) in 2008 [55].

## 2. Characteristics of Aflatoxins

Aflatoxins are chemically derived from difuranocoumarin with a coumarin nucleus-based bifuran group and a lactone ring (AFGs) or a pentanone ring (AFBs and AFMs) [56]. Aflatoxin contamination is highly influenced by environmental factors [57]. Battilani et al., in 2016, reported that the risk of AF contamination can be increased in cereals following an elevation in the rate of temperature for every 2 °C in European countries, including Italy, Spain, Portugal, Turkey, Cyprus, Albania, Bulgaria, and Greece [58]. Moreover, Moretti et al., in 2019 estimated that the risk of AF contamination in maize may be enhanced in Europe because of desired climatic conditions in the next thirty years [59]. Aflatoxin-forming species require temperatures of 25–37 °C and moisture of 80–85% for growth [60]. Therefore, climate changes can alter the temperature and water activity (a_w_) of foods and feeds that affect the expression level of structural (*aflD*) and regulatory (*aflS* and *aflR*) genes and thus induce AF secretion by *Aspergillus* fungi [61,62]. Reverse transcription polymerase chain reaction (RT-PCR) findings showed that the minimum and maximum expression levels of regulatory genes were at the temperatures of 20–37 °C and 28 °C, respectively, highlighting the importance of temperature in the synthesis of AF [62]. Bernáldez et al. in 2017 found that the temperature of 30 °C and the water activity of 0.99 in maize were the optimal conditions for the growth of *A. flavus* according to the analysis of temperature and a_w_ interaction affecting the expression level of *aflR* [63]. In a study by Lv et al., the maximum production of AFB1 was at the temperature of 33 °C and the water activity of 0.96 a_w_ [64]. Gizachew et al. in 2019 reported that the maximum level of AF production was at the temperature of 27 °C and the water activity of 0.90 a_w_ in *A. flavus* and *A. parasiticus* in ground Nyjer seeds [65]. pH is another factor affecting AF production, where maximum and minimum AF production occurs in acidic and basic conditions, respectively [66].

**Figure 1:**
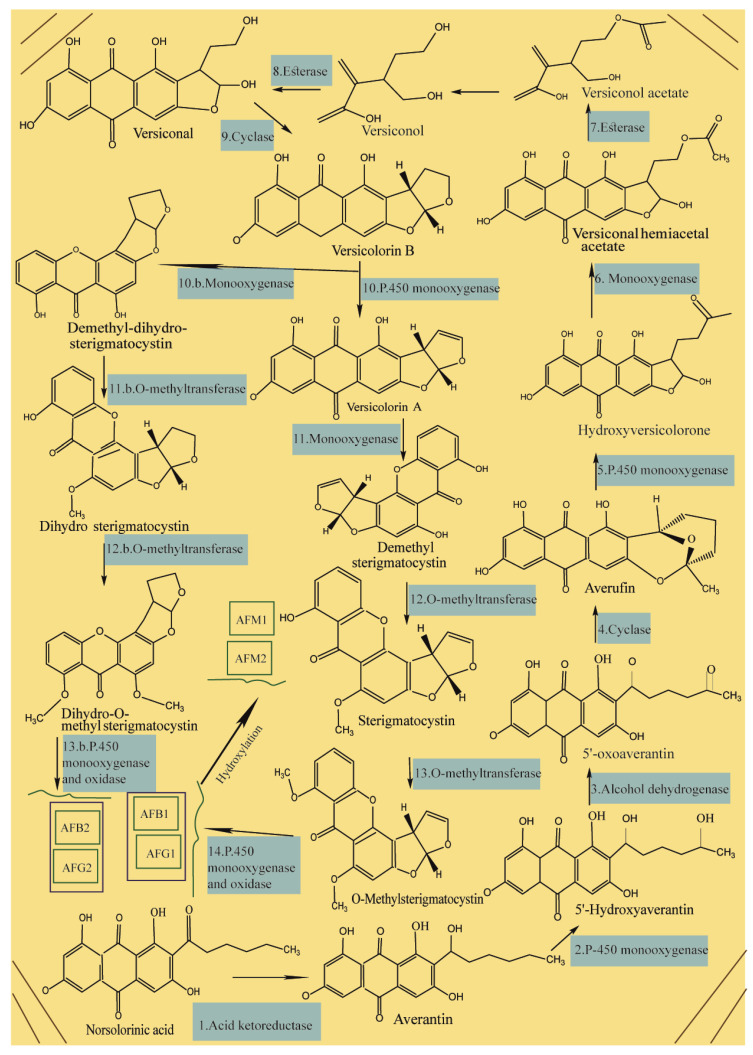
Overview of aflatoxins’ biosynthesis.

During the process of AF biosynthesis in crops by *A. flavus* and *A. parasiticus*, the primary substrate of hexanoyl is converted to polyketide using a polyketide synthase and two fatty acid synthases [67,68,69,70,71], followed by the production of norsolorinic acid anthrone from the polyketide using polyketide synthase and then the conversion of norsolorinic acid anthrone to norsolorinic acid (NOR) as the first stable precursor of AF as shown in Figure 1 [72,73,74,75]. Then, NOR converted to averantin via reductase enzyme, see Figure 1 (1) [76] and then 5′-hydroxyaverantin (HAVN) produced from averantin by monooxygenase enzyme see Figure 1 (2) [77]. Next, the HAVN forms 5′-oxoaverantin (OAVN) using dehydrogenase, see Figure 1 (3), and subsequently OAVN is converted to averufin (AVF) using cyclase, see Figure 1 (4) [78,79,80], followed by the conversion of AVF to hydroxyversicolorone (HVN) via the Baeyer–Villiger reaction, see Figure 1 (5) [81]. After that, versiconal hemiacetal acetate (VHA) is formed via the oxidation of HVN, see Figure 1 (6) that is converted to versiconol acetate (VOAc) and then versiconol (VOH), see Figure 1 (7) [82]; the VOH then uses esterase to produce versiconal, see Figure 1 (8) that is subsequently converted to versicolorin B via cyclase, see Figure 1 (9) [83], followed by the conversion of versicolorin B to versicolorin A and dimethyl-dihydro-sterigmatocystin (DMDHST) as shown Figure 1 (10); then the conversion of versicolorin A and DMDHST to dimethyl-sterigmatocystin (DMST) and dihydro-sterigmatocystin (DHST), respectively, see Figure 1 (11) [84,85,86].

**Figure 2:**
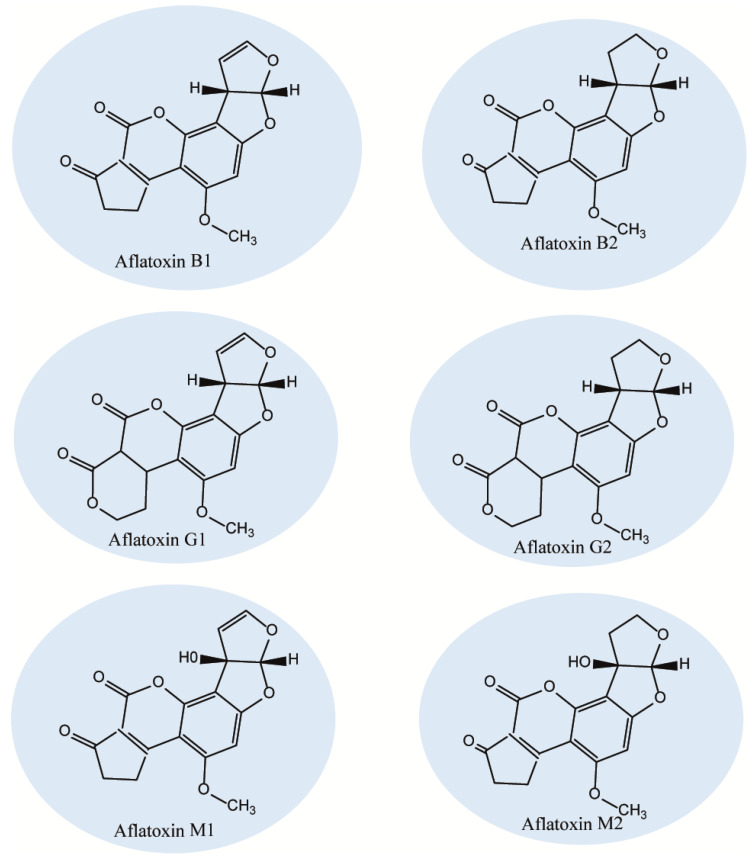
Chemical structures of group 1 carcinogenic aflatoxins.

Next, O-methyltransferases plays central role in the biosynthesis of AFs to convert the intermediates of DMST and DHST to sterigmatocystin (ST) and dihydro-O-methylsterigmatocystin (DHOMST), respectively, as shown in Figure 1 (12) [87]. Afterwards, O-methylsterigmatocystin (OMST) is produced from ST, see Figure 1 (13); finally, OMST and DHOMST lead to the production of AFs, as shown in Figure 1 (13b and 14) [88,89,90,91,92,93,94,95]. Over 20 AF have been identified so far, of which, aflatoxins B1 (AFB1), B2 (AFB2), G1 (AFG1) and G2 (AFG2) have been characterized under UV radiation where AFB1 and AFB2 exhibit a strong blue fluorescence while AFG1 and AFG2 show greenish yellow fluorescence (Figure 2) [96]. According to the evidence, only AFB1/B2 are produced by *A. flavus* and AFB1/B2/G1/G2 are produced by *A. parasiticus*, indicating a difference in the origin of AF [97]. Aflatoxin M1 (AFM1) and AFM2 are not normally present in crops, but the metabolites of these compounds can be separated from the meat and milk products because of consuming AF-B1/AF-B2-contaminated feed [98,99]. The toxicity levels of AF are different according to the following order of toxicity: AFG2 < AFB2 < AFG1 < AFB1 [100]. Aflatoxins are soluble in organic solvents (e.g., chloroform and methanol) and slightly soluble in water, but insoluble in non-polar solutions (e.g., phenyl, cyclohexyl, ethyl, octyl, and octadecyl) [101,102]. Furthermore, the acid pKa of AF as a heat-stable compound is 17.787, with a molecular weight range of 312–346 Daltons [103].

## 3. Contamination of Foods and Feeds

Different factors such as season, post-harvest and management activities, food type and geographical location, have been known to influence AF contamination of a wide variety of foods, feeds thereby causing economic losses [104]. Table 1 reports information on aflatoxins levels in different foods and countries. Analytical methods are also indicated, namely: High Pressure Liquid Chromatography (HPLC); enzyme-linked immunosorbent assay (ELISA), Liquid Chromatography coupled to Mass Spectrometry (LC-MS/MS).

Katsurayama et al., have reported the occurrence of AF in Brazilian rice as less than 14% [117]. They observed that *A. flavus* was observed either in rice or in their cultivation soils from both drylands and wetlands. Initially, five different fungi were isolated and identified on the basis of phenotypic (extrolite and morphology traits), polyphasic and molecular (beta-tubulin gene sequences) properties and then analyzed for AFB1 production, of which, only 17% were able to produce AFB1. Using liquid chromatography-tandem mass spectrometry (LC-MS/MS) and modified quick, easy, cheap, effective rugged, and safe (QuEChERS) techniques, Zhao et al., showed that wheat and cracker samples from Chinese supermarkets had AFB1 contaminations of 18.8% and 8.2%, respectively [118]. Other researchers utilized high-performance liquid chromatography with fluorimetric detection (HPLC-FLD) and competitive enzyme-linked immunosorbent assay (ELISA) techniques to analyze 804 buffalo and cow milk samples for the detection of AFM1, and found a milk sample with AF contamination more than European permissible level (0.05 μg·kg^−1^) [119]. The same methods were employed by Bahrami et al., to evaluate the AFM1 occurrence in traditional dairy products, and the results indicated an AFM1 prevalence of 44.6%, 65.3% and 84.3% in the raw goat, cow and sheep milk, respectively [120]. Granados-Chinchilla et al., assessed food and feed samples for the presence of AF, and the highest AF prevalence was 27.8% and 38.6% for corn ingredients and white corn, respectively [121]. In a study by Heshmati et al., dates, apricots and figs showed a contamination of AFs lower than the maximum limit (4 μg·kg^−1^) reported by the European Union (EU) but dried mulberry exhibited a higher level (4.12 μg·kg^−1^) [122,123]. In a study by Lippolis et al., ginger collected in the rainy season showed AF contamination exceeding the EU limit [124]. Singh and Cotty., reported more than 60% contamination of AFB1 in chilies spice samples [125].

## 4. AF Detection Strategies

The detection of AFs is performed by several conventional methods based on the emission and absorption characteristics, such as liquid chromatography mass spectroscopy (LC-MS) [126], thin layer chromatography (TLC) [127], gas chromatography (GC) [128], high-performance liquid chromatography (HPLC) [129], immunoaffinity column assay (ICA) [130], and enzyme-linked immunosorbent assay (ELISA) [131].

Chromatographic techniques such as HPLC, TLC, LC-MS, and GC are calculated in accordance with the interaction energy of the solute with the stationary phase and the mobile phase. The separated components are distributed between two mobile and stationary phases. The mobile phase, such as supercritical fluids, liquids and gases, penetrate along or through the stationary bed (solid or liquid). The samples needed for analysis are first dissolved in the mobile phase and then used in the stationary phase as a spot. The sample carries along the mobile phase and sorbent, which leads to differential partitions of compounds between stationary and mobile phases in accordance with the moving rate of different components of the sample. The limit of quantification (LOQ) for AFB1, AFB2, AFG1 and AFG2 was reported as 0.5 mg·L^−1^ using the HPLC method in enriched milk and plant-based beverages, meaning it was lower than the maximum EU level [132]. In a study, the levels of AFG1, AFB1, AFG2 and AFB2 were determined in plant-based beverages and enriched milk samples using the LC-MS/MS and HPLC analysis, the results of which the showed a recovery range of 82–104%, an LOQ value of 0.5 mg·L^−1^ and a relative standard deviation of ˂9.7%, suggesting some merits for this method such as a shortened time and reduced cost of data analysis due to ease of use and the need to consume a smaller solvent [133].

The specific antigen-antibody or ligand-receptor bindings make it possible to quantify complexes by immunochemical methods like ELISA and ICA through the absorption of photon energy using the spectrophotometry. Different labels such as radioisotopes, fluorophores and enzymes can be used to amplify the binding process for better signal recognition. In a study by Mohammedi-Ameur et al., the levels of AFM1 was detected by ELISA method, which ranged between 95.59 and 557.22 ng·L^−1^ with a total mean concentration of 71.92 ng·L^−1^ in raw milk, thereby exceeding the USA and EU allowance limit (500 ng·L^−1^ and 0.050 μg·kg^−1^) [134].

Another important approach with regard to AF detection is immunosensor techniques such as electrochemical immunosensors, optical immunosensors, and piezoelectric quartz crystal microbalances that is a biosensor applying antigen or antibody as a biodetector via a signal transducer, such as carbon, gold and graphite, to detect species-specific binding to complement component. In a study by Selvolini et al., an inexpensive and simple approach was used as an electrochemical enzyme-linked oligonucleotide sensor to detect the AFB1 in corn samples, and the findings showed a limit of detection of 0.086 ng·mL^−1^ and dose–response curve of 0.1–10 ng·mL^−l^ [135]. In another study, the aptamer molecular beacon assay was used for the rapid detection of AFB1, which could detect AFB1 spiked in diluted liquor wine, methanol, or corn flour samples with the aid of an aptamer probe [136].

Despite many advantages, the conventional techniques require special skills and are time-consuming methods, so recent efforts have been made to design novel rapid and easy approaches to detect AFs such as hyperspectral imaging (HSI) [137], non-destructive methods based on fluorescence/near-infrared spectroscopy (FS/NIRS) [138] and polymerase chain reaction (PCR).

The molecular structures of substances can be characterized by fluorescence spectrophotometry on the basis of absorption in UV/visible region, but the absorption processes have been employed for some molecules on the basis of various wavelengths of light emission. The molecules can be analyzed and characterized by fluorescence through the emission of energy at specific wavelengths, thus measuring AF (5 to 5000 μg·kg^−1^) within less than 5 min. Rui et al., introduced highly selective surface molecular imprinted polymers (FDU-12@MIPs) approaches as a potent AF adsorbent from AF-contaminated cereals [139]. To this end, the FDU-12@MIPs were first characterized by techniques, including X-ray diffraction (XRD), scanning electron microscopy (SEM), energy dispersive X-ray spectroscopy (EDX) and attenuated total reflection-Fourier transform-infrared spectroscopy (ATR-FT-IR). Subsequently, experiments were continued to analyze rice, peanut, corn, wheat and soybean samples for the presence of AFB1, B2, G1 and G2 using the coupling of HPLC to FDU-12@MIPs. According to the results, an acceptable linear response was obtained for studied AF, ranging from 0.1 to 50 μg·kg^−1^, with an R^2^ ranging from 0.9992 to 0.9996. In this way, the FDU-12@MIPs acted as an impressive adsorbent for the solid-phase extraction to enrich desired AFs in the real samples. Aflatoxin B1 contamination of maize kernels was detected by Kimuli et al., using short-wave infrared (SWIR) hyperspectral imaging (HSI) technique where the maize kernels were categorized by some analytical approaches, including principal component analysis (PCA), partial least squares discriminant analysis (PLSDA) and factorial discriminant analysis (FDA) [140]. Based on the PCA findings, the control kernels were partially separated from kernels contaminated by AFB1 for each variety, but there was no pattern of separation between the pooled samples. The best classification model of PLSDA was obtained by combining first derivative pre-treatments and standard normal variate, with accuracies of 96% and 100% in validation and calibration from Illinois variety, respectively. The best classification model of AFB1 was achieved by FDA on raw spectra, with 100% accuracy in validation and calibration for Nebraska and Illinois varieties. It should be noted that there were poor classification models of AFB1 for the pooled samples when comparing with individual varieties for either PLSDA or FDA models, which can be attributed to the chemical constituent limited variation and also there might have been the introduction of some factors effect such as moisture content, orientation, and year of harvest on these results. The combination of SWIR spectra with spectra pre-treatments and chemometrics predisposed the detection of maize kernels at different AFB1-coated varieties. In accordance with the suggestion of the study, the accuracy of detecting the AFB1 contamination might be affected by the reinforcement of maize kernel constituents like lipid, starch, protein and water in the pooled samples.

PCR technique is able to detect successfully mycotoxigenic fungi present in samples through the co-amplification of species-specific genes and regulatory or structural genes associated with pathways of mycotoxin production. Singh et al., employed real-time PCR to detect AF and found that AFs were present in 53 out of 129 poultry/cattle feed samples [141].

## 5. Toxicity and Health Impacts of Aflatoxins

Aflatoxin-contaminated foods and feeds are associated with health risk for human beings and animals. Aflatoxins have been shown to have different health impacts such as hepatotoxicity [142], mutagenesis [143], carcinogenesis [144], immunosuppression [145], neurotoxicity [146], epigenetic effects [147], reproductive dysfunctions [148] and stunted growth [149]. There have been many studies that scrutinize the mechanisms of these health effects [150,151,152]. Thus, different and strict regulations have been globally implemented to control the contamination of AF in foods and feeds aimed to maintain human and animal health. The maximum permissible levels of AF for human consumption range from 4 to 30 μg·kg^−1^ depending on the food type [153]. The maximum allowed levels of total AFs by the EU is 2 μg·kg^−1^ for AFB1 and 4 μg·kg^−1^ for total AFs [154,155], but 20 μg·kg^−1^ of AFs in the United States [156,157]. The LD_50_ or 50% Lethal Dose value for AFs was 18 mg·kg^−1^ in rats and 0.3 mg·kg^−1^ in rabbits [158].

In a study by Li et al., the dietary 0.6 mg·kg^−1^ of AFB1 inhibited chicken spleen growth via G_0_/G_1_ cell-cycle arrest, as well as reduced mRNA expression of cyclin D1 and elevated CDK6, p21/53 and ATM expression, suggesting that AFB1 induced G_0_G_1_ phase arrest through activated ATM-p53-p21-cyclin D/CDK6 route in the splenocytes [159]. Chen et al. investigated whether the toxicity of AFB1 on Leydig cells could be attributed to the enhancement of ROS generation, the prevention of T-biosynthesis gene expression, the reduction in Leydig cell count, and induction of cell apoptosis via AMPK/mTOR-mediated suppression of autophagic flux [160]. In an in vitro study, Liu et al., reported genotoxic impacts induced by AFB1 and MC-LR combinative exposure in hepatocytes through oxidative stress and DNA base excision repair genes [161]. AF-contaminated feeds (0.3 and 0.6 mg·kg^−1^) among male broilers could increase the apoptotic splenocytes through elevated oxidative stress [162]. AFB1-induced hepatocarcinogenesis can be developed by the impacts of aldehydes production following the formation of hepatic AFB1 metabolism-induced LPO, as some of these effects are the induction of a hepatic prone to mutagenesis induced by DNA damage, DNA repair prevention, mutated codon 249 of p53 gene, DNA damage induction and LPO cycle propagation [163]. Frequent consumption of AFB1 in adult male rats impaired the hypothalamic regulation of neuropeptides in feeding behaviour [164]. Peng et al. reported that AFB1 could influence apoptosis and the expression of Bax, Bcl-2, and Caspase-3 in the thymus and bursa of fabricius in broiler chickens [165].

## 6. Methods of Aflatoxin Detoxification

High AF detoxification resistance to common treatment strategies such as pasteurization and sterilization have been reported, therefore necessitating the development of effective physical, chemical and biological approaches to control AF [166,167,168,169,170].

**Figure 3:**
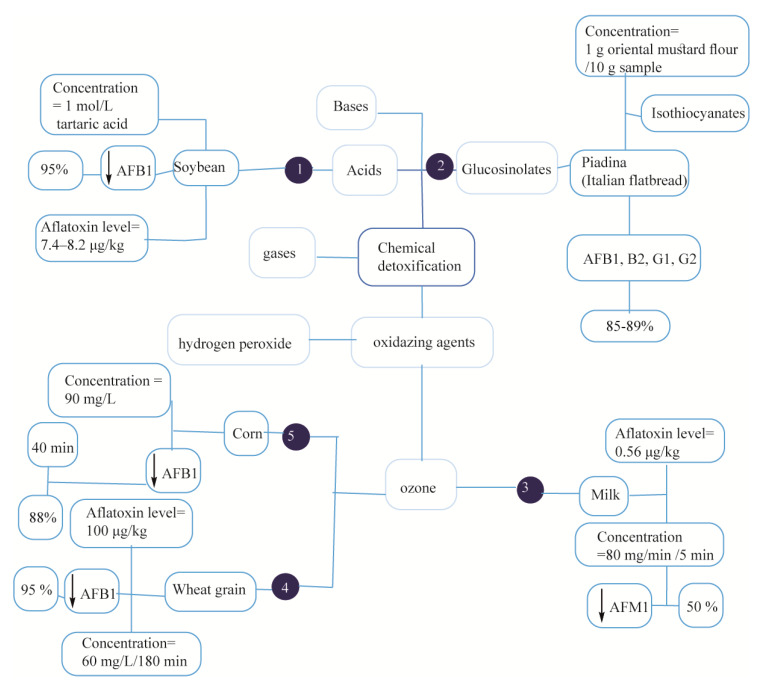
Overview of chemical detoxification methods. (1, [171]), (2, [172]), (3, [173]), (4, [174]), (5, [175]).

Aflatoxin detoxification may occur through the degradation of its structure using different gases or chemical agents that oxidize (e.g., hydrogen peroxide or ozone) or hydrolase (e.g., aldehydes, bases or acids) or thermal treatment. In the hydrolysis method of detoxification, acidic and alkaline conditions are able to open the lactone rings of AF to form a water-soluble compound called beta-keto acid that is easily removed from the sample by rinsing with water (Figure 3). Aflatoxin B1-contaminated soybean (7.4–8.2 μg·kg^−1^) treated by tetraic acid for 18 h showed 95% detoxification using High Performance Liquid Chromatography with Fluorescence Detection (HPLC-FLD) as a quantitative analysis as reported in Figure 3 (1), and in Figure 3 (6) [171]. Saladino et al., reported 89% detoxification of AFB1 in Italian piadina exposed to isothiocyanates with antimicrobial properties, thereby inhibiting *A. parasiticus* growth on the samples as illustrated in Figure 3 (2) [172]. Mohammadi et al., observed a 50% AFM1 detoxification (0.56 μg·kg^−1^) in milk samples using a chemical detoxification method via 80-mg·min^−1^ ozonation for 5 min, see Figure 3 (3) [173]. A 60 μmol·mol^−1^ ozonation of AFB1-contaminated wheat for 180 min led to a 95% detoxification as illustrated in Figure 3 (4) [174]. A 40-min ozonation of the AFB1-contaminated corns with 13.5% of moisture content reduced the AFB1 level up to 9.9 μg·kg^−1^ from 83 μg·kg^−1^ as shown in Figure 3 (5) [175]. Rastegar et al., investigated the removal of AFB1 by roasting with lemon juice and/or citric acid in naturally contaminated pistachio nuts [176]. They reported a 93.1% decrease in AFB1 level after roasting pistachio nuts (50 g) in the presence of water (30 mL), lemon juice (30 mL) and citric acid (6 g) at a temperature of 120 °C for an hour. They also reported a 49% AFB1 level decrease following an alteration of citric acid and lemon juice concentration. Therefore, there was a synergistic impact between lemon juice/citric acid concentration and heating on AFB1 degradation. Rushing and Selim converted over 71% AFB1 to its detoxified form, AFB_2_a, in contaminated feed through a similar citric acid treatment [177]. Chen et al. employed the ozonation technique to detoxify 65.9% and 65.8% of AFB1 and total AFs in the peanuts, respectively, and stated that the exposure time and the ozone concentration were two factors affecting the detoxification of AFs [178]. Aflatoxins can be attenuated by chemical degradation of nutrients in spite of some disadvantages, such as the high cost, and low aesthetic quality of treated foods and feeds.

**Figure 4:**
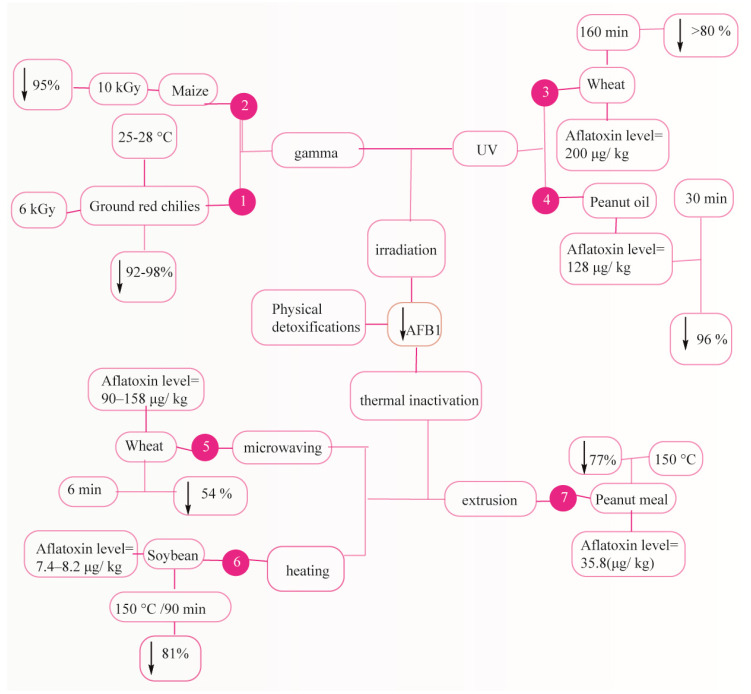
Overview of physical detoxification methods. (1, [179]), (2, [180]), (3, [181]), (4, [182]), (5, [183]), (6, [171]), (7, [184]).

Thermal inactivation (e.g., microwaving, extrusion, and heating), irradiation ultraviolet light (UV) and gamma radiations), and adsorption agents (e.g., bentonite, hydrated sodium calcium aluminosilicate (HSCAS)) are the most prevalent physical techniques to detoxify AF (Figure 4).

High temperatures of between 237 and 306 °C are heating methods of detoxification. Numerous researchers recruited gamma radiation decontamination called as a cold process to extend food shelf life by declining microbial density. Mycotoxins are significantly degraded by effective doses of gamma radiation. Iqbal et al. reported 92% to 98% detoxification of AFB1 in chili samples exposed to 6-kGy dose of gamma (γ) radiation, see Figure 4 (1) [179]. Another study showed about 94.5% AFB1 detoxification in 50 μg·kg^−1^ maize feeds following 10-kGy dose of γ irradiation, see Figure 4 (2) [180]. Ghanghro et al. found 82% to 90% detoxification of AFB1 wheat grain (200 μg·kg^−1^) following 160-min UV radiation as shown in Figure 4 (3) [181]. Mao et al., observed a 96% detoxification of AFB1 peanut oil (128 μg·kg^−1^) following 30 min UV irradiation using Ultra Performance Liquid Chromatograph-Thermo Quadrupole Exactive Focus mass spectrometry/mass spectrometry (UPLC-TQEF-MS/MS analysis) as shown in Figure 4 (4) [182]. In another study, the effect of microwave heating wheat samples at 160 °C for 6 min resulted in a 54% reduction in AFB1 as shown in Figure 4 (5) [183]. In a study by Zheng et al., AFB1-contaminated peanut meals were exposed to extrusion cooking process, and finally the results showed an AFB1 degradation rate of 77.6% ± 2.2% at a temperature of 150 °C. (Figure 4 (7)) [184]. Kanapitsas et al. observed a 65% AFB1 reduction in raisin samples following a 10kGy gamma irradiation [185]. Wang et al., reported that 15-s pulsed light treatment decreased AFB1 and AFB2 levels up to 90.3% and 86.7%, respectively, in rice bran samples gathered from the Farmers’ Rice Cooperative (West Sacramento, CA, USA), whereas 80-s treatment decreased the AFB1 and AFB2 levels up to 75.0% and 39.2% in rough rice, respectively [186]. Despite several physical detoxification methods, these approaches eliminate the AFs in part and are time-consuming.

In the adsorption techniques, toxin-absorbent binding in the gastrointestinal tract can decrease the content of mycotoxins, and proper positioning of functional groups and polarity can be effective for better adsorption of AF. The main adsorbing compounds are synthetic polymers (polyvinyl pyrrolidone, cholestyramine, cellulose, polysaccharides, peptidoglycans, glucomannans, and alumino (hydrated sodium calcium aluminosilicate [HSCAS], bentonite, clay, sodium and calcium aluminum silicates). Moussa et al., in Egypt, evaluated the efficacy of calcium bentonite clay and kaolin on AFM1-contaminated raw milk samples (50 ng·L^−1^) collected from dairy shops [187]. They treated the samples with different concentrations of calcium bentonite clay and Kaolin for the detoxification of AFM1, and then detected the AFM1 level by ELISA. According to their findings, the mean AFM1 concentration in raw milk samples was 10.7 ± 0.89 ppb, indicating that the raw milk samples exceeded the EU permissible limits (50 ng·L^−1^) and Egyptian standards (50 ng·L^−1^) of AFM1 in milk; the rate of AFM1 detoxification was between 86.1% and 97.7%. In a study, highly active sodium bentonite (SB) soil (SB-E) was used to absorb AF, the results of which showed the maximum binding capacity of these biological adsorbents to AF at pH values of 6.5 and 2, with high enthalpy (-H) and confirmed their safety approved by Hydra bioassay [188].

**Figure 5:**
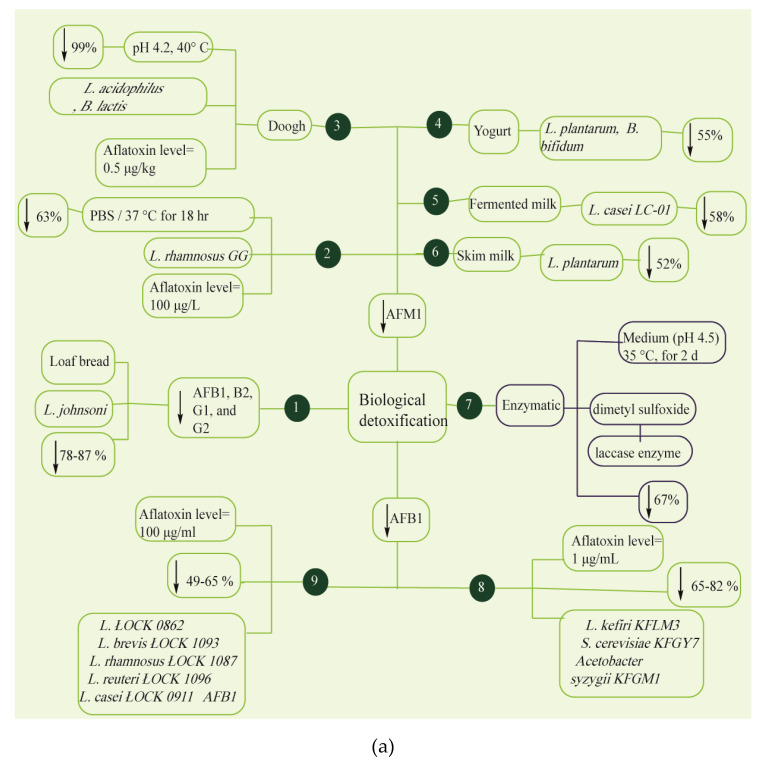

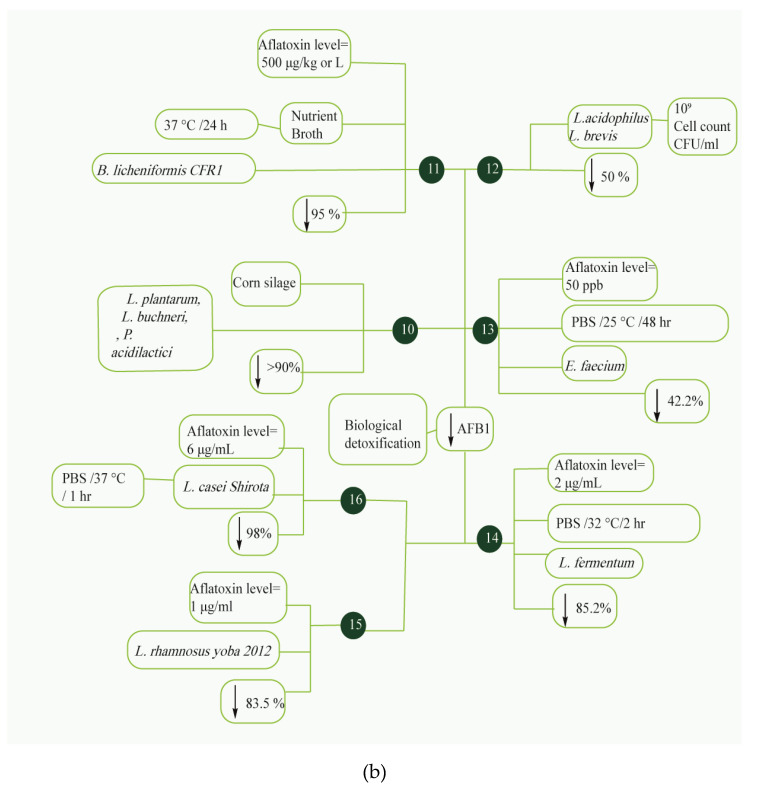
Overview of biological detoxification methods. (**a**): (1, [210]), (2, [211]), (3, [212]), (4, [213]), (5, [214]), (6, [215]), (7, [216]), (8, [217]), (9, [232]); (**b**): (10, [218]), (11, [219]), (12, [220]), (13, [221]), (14, [222]), (15, [223]), (16, [224]).

**Figure 6:**
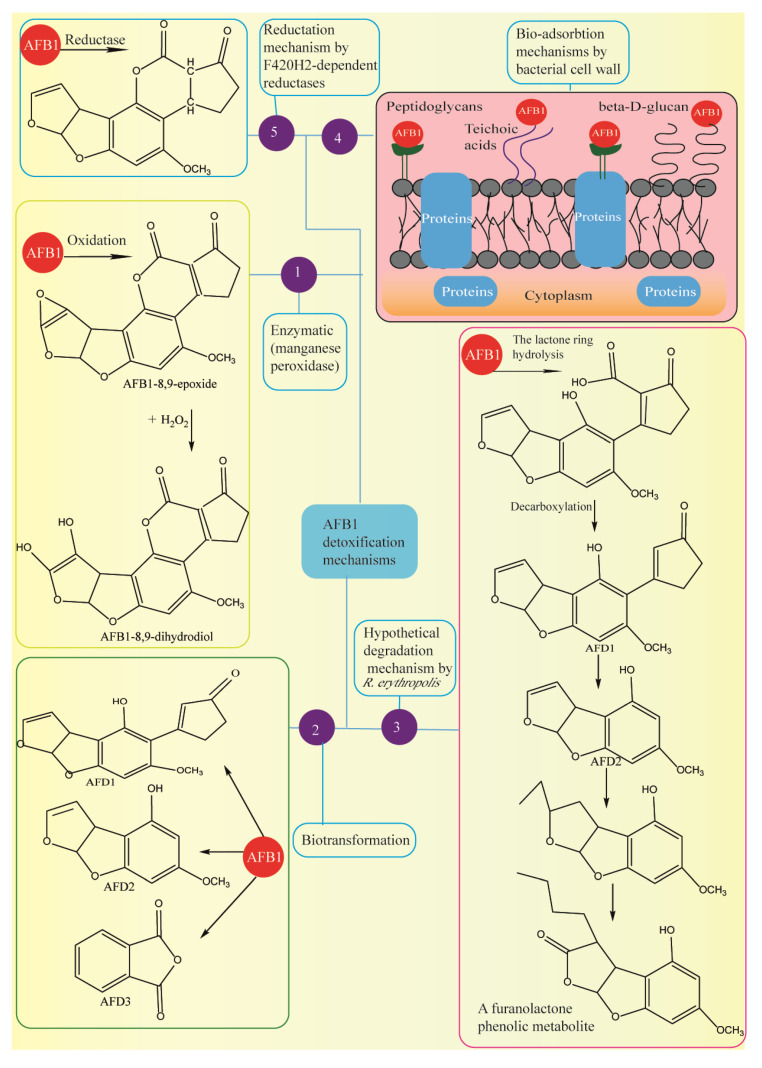
Overview of aflatoxins detoxification mechanisms. (1, [235]), (2, [230]), (3, [231]), (4, [224,225,226,227]), (5, [236]).

The application of enzymes and microorganisms in AF bio-detoxification is a good alternative to conventional techniques in the food industry [189,190,191,192,193,194,195,196,197,198,199,200,201,202,203,204,205] (Figure 5a,b). There are two mechanisms for AF detoxification by microbial methods, these are: cell wall component adhesion and microbial enzymes. Lactic acid bacteria (LAB) and yeast strains are utilized in fermented food products and beverages as starters due to their ability to detoxify AFs. Aflatoxin bio-absorption mechanisms of *Lactobacillus*, fungi and other bacteria have been reported by several authors [206,207,208,209]. Saladiano et al. reported 84.1–99.9% reduction in AF levels in contaminated bread due to LAB and yeast fermentation for 3–4 days, see Figure 5a (1) [210]. High-Performance Liquid Chromatography analysis exhibited 63% detoxification of AFM1 in milk (100 μg·kg^−1^) through non-covalent electrostatic binding such as Van der Waals forces and hydrogen bonds because of the inoculation of *L. rhamnosus* GG (5 × 10^8^ CFU mL^−1^) at a temperature of 37 °C for 18 h, see Figure 5a (2) [211]. Sarlak et al. removed AFM1 from doogh by adding 9 log CFU·mL^−1^ of *L. acidophilus* at pH 4.2 and observed less reduction in non-viable (heat-killed) bacteria than in viable bacteria, see Figure 5a (3) [212]. The co-administration of LAB strains and inulin led to 55% detoxification of AFM1 in yogurt samples as illustrated in Figure 5a (4) [213]. *L. casei* LC-01 reduced AFM1 levels by 58% in the fermented milk (Figure 5a (5)) [214]. In the in vitro study of Panwar et al., 24-h incubation of probiotic lactobacilli in AFM1-contaminated skim milk reduced AFM1 levels by up to 52% during digestion tests as shown in Figure 5a (6) [215]. Zeinvand-Lorestani et al., reduced AFB1 levels by 67% in the presence of laccase enzyme after two days, see Figure 5a (7) [216]. Kefir microorganisms decreased AFB1 levels by 82% by binding to AFB1 (1 μg·kg^−1^) as shown in Figure 5a (8) [217]. Ma et al., used 10^9^ cfu·g^−1^ of corn silage bacteria and reached AFB1 levels to 0.35 µg·kg^−1^ within three days incubation period, see Figure 5b (10) [218]. A study by Rao et al. achieved the microbial AFB1 degradation rate of 94.7% using *Bacillus licheniformis CFR1* that had been confirmed via Electron spray ionization-Mass Spectrometry (ESI-MS), HPLC, High-Performance Thin Layer Chromatography (HPTLC) analysis, see Figure 5b (11) [219]. In a study by Sadeghi et al., *L. acidophilus* and *L. brevis* caused 50% detoxification of AFB1 after 24 and 48 h of incubation, see Figure 5b (12) [220]. In an in vitro study by Fernandez et al., the strains of *E. faecium* isolated from dog stool samples could eliminate AFB1 by 42% after 48 h of incubation, see Figure 5b (13) [221]. Binding capacity of *L. fermentum* led to 85% detoxification of AFB1 in the media after two hours incubation period, see Figure 5b (14) [222]. High-Performance Liquid Chromatography analysis showed 1000-fold detoxification of AFs due to the starter culture with *L. rhamnosus yoba* (10^8^ cfu·g^−1^), see Figure 5b (15) [223]. According to findings, *L. casei* showed 98% AFB1 binding (4.6 µg·mL^−1^) through bioabsorption process across cell wall peptidoglycan and polysaccharides (Figure 5b. 16) [224]. Others reported that AFB1 was detoxified by *L. rhamnosus strain GG* through binding to cell surface proteins as shown in Figure 6 (4) [225]. In a study by Hernandez- Mendoza et al., *L. reuteri strain* NRRL14171 and *L. casei strain Shirota* were able to show AFB1 detoxification activity by binding to teichoic acids and peptidoglycans, see Figure 6 (4) [226]. Yiannikouris et al., demonstrated the central function of (1→3)-β-D-glucans conformation of the bacterial cell wall in the interactions with AFB1 via intermolecular hydrogen bonding and Van der Waals force, see Figure 6 (4) [227].

Rabie et al. found a 78% reduction in AFM1 in milk by Lactobacillus acidophilus and Bifidobacterium lactis after one-day incubation [228]. Martínez et al. observed a decrease in AFM1 in milk through the bio-degradation and bio-adsorbtion mechanisms in Pediococcus pentosaceus and Kluveromyces marxianus [229]. In a study by Samuel et al., Pseudomonas putida could tolerate the exposure of AFB1 (0.2 mg·mL^−1^) in the medium [230]. Based on the findings of FTIR, GCeMS, HPLC, TLC and UV spectrometry analysis, AFB1 biotransformation to AFD1, AFD2, and AFD3, as shown in Figure 6 (2) during 24-h incubation modulated AFB1 ring lactone and furan and declined the toxicity. In another study, S. aureofaciens ATCC 10762, Rhodococcus erythropolis ATCC 4277 and Streptomyces lividans TK 24, three species of Actinomycete, were co-cultured to degrade AFB1 in a liquid medium, see Figure 6 (3) [231]. The results showed that AFB1 was detoxified by these strains through various mechanisms; for example, the TLC method reported AFB1 degradation via R. erythropolis through the lactone cleavage. According to an in vitro study by Chlebicz and Śliżewska, the level of AFB1 was decreased by S. cerevisiae and Lactobacillus sp. by up to 65% and 60%, respectively, as illustrated in Figure 5a (9) [232].

Liu et al. reported the detoxification of AFB1 in cottonseed meal by *Cellulosimicrobium funkei* bacterium [233]. In a study by Hontanaya et al., dry mustard flour glucosinolates decreased AFs in the nuts and fruits by 88–89% [234]. In a study, AFB1-contaminated foods were detoxified by the manganese peroxidase (MnP) extracted from Phanerochaete sordida YK-624, a white-rot fungus, see Figure 6 (1) [235].

The efficiency of AFB1 degradation was 86.0% after 48 h. The analysis of HR-ESI-MS and H-NMR techniques demonstrated that the oxidization of AFB1 initially generated AFB1-8,9-epoxide in the presence of MnP, and then the hydroxylation led to the production of AFB1-8,9-dihydrodiol. According to other reports, the reductases from mycobacteria were able to detoxify AFB1 through the AFs α,β-unsaturated ester moiety reduction, catalyzing the deazaflavin cofactor F_420_H_2_, as shown in Figure 6 (5) [236]. The growth of fungus *Pleurotus ostreatus* on various agricultural residues leads to the formation of ligninolytic enzymes involved in the detoxification of AFB1. Accordingly, Das et al. co-cultivated AFB1-contaminated rice straw with *P. ostreatus*, the result of which was 89% detoxification of AFB1 [237]. The results of a study showed 100% prevention of AF formation in the presence of natural powdered pomegranate peels (at the concentrations of 5%, 10%, 20%, combined with inoculated rice, *w*/*w*) for the four month-storage of rice at the moisture of 18% and the temperature of 25 °C, whereas lemon peels had inhibitory effect during three months [238]. In a recent study, Neem leaves, which are agricultural residues by-products, inhibited AF formation within two and four months when used in maize and wheat products, respectively [239].

## 7. Conclusions

Aflatoxin contamination of foods and feeds results in economic losses and affects human and animal health, either directly or indirectly. Inadequate knowledge in this area highlighted the necessity of investigations into the chemical properties and biosynthetic processes of AFs and various mechanisms of their detoxification, also considering possible natural agents against the proliferation of field pests for the crops [240]. Numerous studies have been conducted recently to control these toxins, but many are not yet developed at the commercial scale. Accordingly, further research is recommended to focus on field-applicable new technologies for the control of AFs with the aim of protecting human and animal food/feed safety and health. In general, all people involved in commodity value chains should consider AF control measures to promote food safety, increase awareness about public health and prevention, raise economic benefits, and decrease costs.

## Figures and Tables

**Table 1:** Prevalence and levels of aflatoxins in different foods from different countries.

Aflatoxin Type	Food/Feed Type	Area of Origin	Sample Size	Mean and/or Median Levels	Range Levels	Analysis Method	Reference
**AFB1**	Black tea	Pakistan	+76%	0.11 and 16.17 μg·kg^−1^	0.08–8.24 μg·kg^−1^	HPLC	[105]
**AFB1**	Chinese condiment (Doubanjiang)	China	+34%	4.78 ± 0.16 μg·kg^−1^	1.26–16.41 μg·kg^−1^	ELISA	[106]
**AFB1**	Peanuts	Zambia	+44%	0.45 μg·kg^−1^	0.015–46.60 μg·kg^−1^	HPLC	[107]
**AFB1**	Spices	Italy	+15%	0.30 μg·kg^−1^	0.59–5.38 μg·kg^−1^	HPLC	[108]
**AFB1**	Maize flour	Turkey	+66%	0.20 μg·kg^−1^	0.041–1.12 μg·kg^−1^	HPLC	[109]
**AFB1**	Maize	Serbia	+57%	11.4 ± 14.5 μg·kg^−1^	1.3–88.8 μg·kg^−1^	HPLC	[110]
**AFM1**	Milk	Portugal	+27%	23.4 ± 24.0 ng·L^−1^	0.005–0.069 μg·kg^−1^	ELISA	[111]
**AFM1**	Milk	Indonesia	+95%	216 ng·L^−1^	24–570 ng·L^−1^	ELISA	[112]
**AFM1**	Milk	China	+80%	23.7 ng·L^−1^	5.1–104.4 ng·L^−1^	ELISA and LC-MS/MS	[113]
**AFM1**	Milk	Lebanon	+58%	0.035 µg·L^−1^	0.011–0.440 μg·kg^−1^	HPLC	[114]
**AFM1**	Infant formulae	Mexico	+20%	40 ± 99 ng·L^−1^	40–450 ng·L^−1^	HPLC	[115]
**AFB1, AFB2, AFG1, and AFG2**	Household maize	Kenya	+100%	62.5 μg·kg^−1^	2.14–411 μg·kg^−1^	UHPLC	[116]
